# Can primary care nurse administered pelvic floor muscle training (PFMT) be implemented for the prevention and treatment of urinary incontinence? A study protocol

**DOI:** 10.12688/f1000research.2-47.v1

**Published:** 2013-02-13

**Authors:** Sue Child, Alice Bateman, Joanna Shuttleworth, Christian Gericke, Robert Freeman

**Affiliations:** 1 Peninsula CLAHRC, National Institute for Health Research, The University of Exeter Medical School, Exeter, EX2 4SG, UK; 2 Peninsula CLAHRC, National Institute for Health Research, Plymouth University, Peninsula College of Medicine and Dentistry, Plymouth, PL4 8AA, UK; 3 The Wesley Research Institute, Brisbane, 4066, Australia; 4 Department of Obstetrics and Gynaecology, Plymouth Hospitals NHS Trust, Plymouth, PL6 8DH, UK

## Abstract

**Background: **We aim to evaluate if Pelvic Floor Muscle Training (PFMT) delivered in primary care results in fewer referrals to secondary care for urinary incontinence (UI), thereby reducing the number and associated costs of surgical procedures for UI.

**Methods / design:** The study will consist of two populations – a prevention group and a treatment group who will both be offered PFMT in primary care. The prevention group will consist of parous women aged 25-64 attending for a routine cervical smear. Their pelvic floor will be assessed using the Modified Oxford Scale (MOS) and a baseline data form will be completed that asks about the frequency and associated bother of urine leakage. From the answers given, the group will be subdivided into two groups. The first (prevention) group will be subdivided into a primary prevention arm (no symptoms of urinary incontinence and pelvic floor strength ≤2 on MOS) and a secondary prevention arm (women reporting symptoms of urine leakage irrespective of MOS). The second (treatment) group will be women of any age who may or may not have had a vaginal birth presenting to their GP with UI. Semi-structured, in-depth interviews will be conducted with a subset of patients and staff with the aim of identifying barriers and facilitators in delivering PFMT in primary care.

**Discussion:** A recently completed community study showed good outcomes with practice nurse delivery of PFMT. We suggest if this were to be implemented more widely it would reduce the need for referral to secondary care. We believe that this study will show whether implementing a package of PFMT delivered in primary care can treat as well as prevent UI and will also be helpful in exploring the benefits / drawbacks of such implementation, thus providing lessons for implementation in other Primary Care Trusts (PCTs).

## Study rationale

Urinary incontinence (UI) is a distressing and disabling condition as it involves the involuntary leaking of urine from the bladder. It is estimated that 10 million women in the UK suffer with UI ^1^ at a cost to the National Health Service of £594 million per year ^2^. Prevalence rates vary depending on age and severity. Hunskaar et al., estimate prevalence is between 20–30% for young adult women (18–44), 30–40% for middle age women (45–49) and 30–50% for elderly women (≥60 years) ^1^. One of the main forms of UI is stress urinary incontinence (SUI), which can occur with coughing, sneezing or exercise.

The pelvic floor muscles are often described as a ‘sling’ supporting the pelvic organs and are located beneath the bladder and rectum. They are involved in the process of storing and passing urine. These muscles often become weakened as a result of childbirth and as a result the individual is much more likely to develop some kind of UI. Worldwide, the World Health Organisation (WHO) report that one third of women have urinary incontinence after childbirth ^3^. Supervised pelvic floor muscle training (PFMT) or ‘pelvic floor exercise’ aims to strengthen these muscles to help treat and prevent UI. Evidence suggests the more the pelvic floor is exercised the better the result ^3^.

It is known that the pelvic floor muscles can be difficult to identify for individual women and that supervision is required to enable them to do pelvic floor exercises properly ^4^. In addition, it has been shown that 30% of women asked to do so are unable to contract their pelvic floor properly ^4^. Even in young, fit, nulliparous women one in ten are unable to contract the pelvic floor to any extent ^4^, and where exercise is undertaken more than a quarter use a technique that could potentially promote incontinence rather than contract the pelvic floor muscle ^5^. Once learnt properly, pelvic floor exercises can produce favourable results that can persist in many women for at least ten years ^6^. At a population level we know little about the status of the pelvic floor or about the number of women who are able or unable to perform pelvic floor contractions or how many do pelvic floor exercises regularly.

It has been recommended by NICE guidelines that the first line treatment for urinary incontinence should be provided in primary care ^7^. The most appropriate form of PFMT is supervised training given by a physiotherapist or a specialist continence nurse, who will have greater in-depth knowledge and expertise. However, there are insufficient numbers of trained staff to provide this for all women either in primary or secondary care.

Work already completed by a research team at Derriford Hospital, Plymouth has demonstrated that a practice nurse can deliver PFMT with outcomes comparable to those of a specialist nurse ^8^. Practice nurses are in an ideal position to provide this treatment as most undertake cervical smears, which is an appropriate time to assess the pelvic floor and offer PFMT if necessary. We believe that as most practice nurses are more accessible to the woman and her family, it is likely that compliance with training could be better than that given by someone with whom the woman is unfamiliar, for example a continence advisor or a physiotherapist. The results from Waterfield’s study showed good outcomes with practice nurse delivery of PFMT and if this were to be implemented widely would reduce the need for referral to secondary care ^8^.

Evidence suggests that 50% of women with urinary incontinence referred to secondary care have not received PFMT ^9^. Throughout Plymouth Primary Care Trust – known more widely as NHS Plymouth (the body that commissions primary care throughout Plymouth), over 50 referrals were returned to the referring GP during the first six months of 2011 because patients had not received PFMT before referral. If PFMT can be provided in primary care there is evidence to indicate that this can prevent as well as treat urinary incontinence and potentially reduce the need for surgery ^8^.

## Methods/design

### Implementation process (populations)

**Figure 1.**
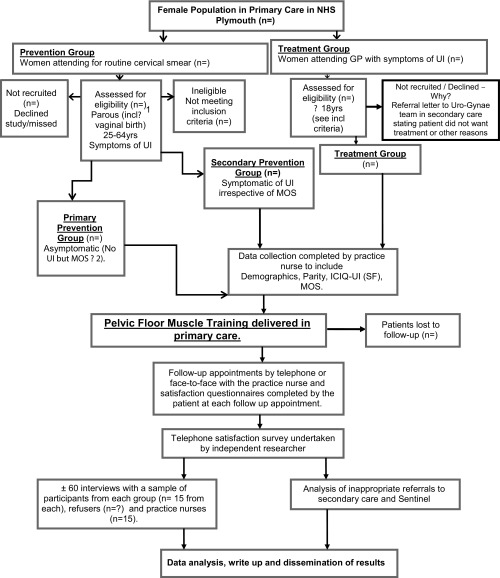
Can primary care nurse administered pelvic floor muscle training (PFMT) be implemented for the prevention and treatment of urinary incontinence? Flow diagram of study design.

The study will comprise of two populations – a prevention group and a treatment group (see Figure 1 for a flow diagram of the study design). Women will be allocated to either of these two groups (following gathering of baseline data and assessment of their pelvic floor muscle strength) as follows:

1. **Prevention group** - Women aged 25–64 years who have had at least one vaginal birth (i.e. parous women) attending their GP surgery for a routine cervical smear. This group will be further subdivided into a primary prevention arm and a secondary prevention arm following the information provided from the completion of the baseline data form at the time of the smear:

a) **Primary prevention arm** - Women with no symptoms of urinary incontinence but a weak Modified Oxford Scale score (≤ 2).

b) **Secondary prevention arm** - Women symptomatic of mild (i.e. not bothersome) urinary incontinence who have not previously sought medical treatment from their GP for UI irrespective of Modified Oxford Scale score.

2. **Treatment group** - Women over the age of 18 years who may or may not have experienced a vaginal birth presenting at their GP surgery with urinary incontinence.

### Inclusion criteria

Women (aged over 18) from the primary prevention group who have a score of two or less on the Modified Oxford Scale and all women from the secondary prevention and treatment groups irrespective of their Modified Oxford Scale score will be eligible for the study.

### Exclusion criteria

Women under 18, and those over 18, but unable to speak English or who have special communication needs will be excluded from the study. Patients who have evidence of urinary infection, pelvic pain, blood on urinalysis, visible prolapse, pelvic mass, voiding difficulties or other abnormalities will also be excluded.

### Implementation process (training of professionals and localities)

NHS Plymouth has 41 GP practices in 5 geographic localities with around 230 fully trained smear takers who are mostly practice nurses. They undertake approximately 16,000 cervical smears every year. In order to implement the delivery of PFMT in primary care, cervical smear takers will need to be trained to undertake pelvic floor assessment and deliver PFMT to women tailored to individual need. Such training will include gaining an understanding of the principles involved in pelvic floor muscle assessment including digital vaginal palpation known as the Modified Oxford Scale score. At the end of this training they will be able to assess pelvic floor muscle condition, be able to advise the patient on an individual programme of exercise and to set targets for that exercise.

Training will be staggered between localities in order for subsequent evaluation to take place on a rolling basis. Two localities with differing social class demographics will be chosen for initial training roll-out. By doing this, it may be possible to ascertain whether barriers and facilitators relating to the delivery of PFMT in primary care differ by socio-economic status. For the purposes of this evaluation, socio-economic status will be measured by occupation and postcode.

### Implementation evaluation

The evaluation of implementation will be undertaken by the Collaboration for Leadership and Applied Health Research and Care for the South West Peninsula (PenCLAHRC). The study will assess the following outcome measures.

### Primary outcome measure

***Reduce cost by fewer referrals to secondary care***. For the treatment group, to assess whether delivering PFMT in primary care results in fewer referrals to secondary care, thereby reducing the number and associated costs of surgical procedures for UI. This is in keeping with the QIPP agenda (a large scale transformational programme for the NHS, involving all staff aimed at improving the quality of care the NHS delivers whilst making up to £20 billion of savings by 2015 to be reinvested in frontline care).

### Secondary outcome measures

***Patient satisfaction***. As a marker of service quality, patient satisfaction of practice nurse delivered PFMT will be assessed at the end of PFMT training through the completion of a modified Royal College of General Practitioners (RCGP) Short-Form Patient Satisfaction Questionnaire (PSQ-18) by all women who have been recruited to PFMT training.

### Compliance and default rates

Compliance and default rates with PFMT training in the treatment group will be monitored against both sub-groups in the prevention group through attendance at follow-up consultations. Each woman will be offered at least one follow-up consultation three months after the commencement of PFMT training either by telephone or face-to-face depending on preference. Training will be reviewed and further measures for improvement will be suggested if necessary. Follow-up data will be collected using a data collection sheet that will repeat the modified questions from the International Consultation on Incontinence Questionnaire (ICI-Q) completed at baseline (see Questionnaire files). The responses will allow us to ascertain if there have been any changes to the symptoms of UI and indicate levels of compliance with PFMT training offered at baseline. If the woman would like to continue with follow-up after her first follow-up consultation then she will be allowed to do so for a maximum of three further occasions at three monthly intervals.

Data collection sheets (baseline and follow up) Data collection sheets that repeats the modified questions from the International Consultation on Incontinence Questionnaire (ICI-Q), completed at baseline and follow up

### Clinical measures

Clinical outcomes will examine whether patient-reported symptoms of UI are improved after a course of primary care nurse-delivered PFMT, through the completion of a modified ICI-Q Short Form (SF) questionnaire at baseline and at each follow-up (see Questionnaire files). This data will also allow us to establish the prevalence of low grade urinary incontinence and the general pelvic floor muscle condition in the female population in Plymouth.

Semi-structured, in-depth interviews will be carried out by an experienced qualitative researcher with a sample of patients and practice nurses, receiving or delivering PFMT in primary care in order to identify facilitators and barriers to implementation. It is hoped that 60 interviews will be conducted in total, with as far as possible equal numbers from all three groups. Women who refuse PFMT training will also be approached for interview in order to further understand barriers and facilitators to delivering PFMT in primary care.

Once interview transcripts have been analysed, a matrix will be designed which will enable us to ascertain key themes and create an understanding of the individual and group coding. The interviews will add depth to the analysis of quantitative (baseline and follow-up) data.

## Discussion

The implementation of PFMT in primary care in Plymouth will aim to reduce the number of referrals to secondary care, thereby reducing costs to the NHS. Our evaluation will provide routinely collected data at baseline and at follow-up enabling an assessment of the impact of implementing this type of service change in the NHS. In addition, the qualitative analysis will allow us to ascertain why such a service implementation might be successful and how both practice nurses and patients perceive the benefits and drawbacks of such a change. Our expectation is that the implementation of PFMT in primary care will reduce referrals to secondary care and, through the process of accepting a package of PFMT, empower women to improve their own self-care. It will be interesting to see if these expectations are realised.

## Ethical approval

After advice from the National Research Ethics Service South-West (Bristol), UK, the quantitative (baseline and follow-up data) and qualitative evaluations planned for this evaluation of implementation do not require full NHS ethical approval as it is an evaluation of a change in service. However, all interview respondents (both patients and professionals) will be volunteers. Written, informed consent will then be gained using a standardised consent form. Confidentiality will be emphasised at the outset and interview transcripts will be anonymised.
