# Supplementary material for: Can primary care nurse administered pelvic floor muscle training (PFMT) be implemented for the prevention and treatment of urinary incontinence? A study protocol
Source: F1000Res. 2013 Feb 13;2:47. [Version 1] doi: 10.12688/f1000research.2-47.v1 (PMC3752732; doi:10.12688/f1000research.2-47.v1)
Supplement: Data collection sheets (baseline and follow up) — Data collection sheets that repeats the modified questions from the International Consultation on Incontinence Questionnaire (ICI-Q), completed at baseline and follow up [file f1000research-2-1176-s0000.tgz › Data_Collection_Sheet__baseline_.pdf]

Data Collection Sheet (baseline) – to be completed in consultation with the patient.

CONFIDENTIAL

Please insert your practice stamp

1. **Background Information** **Date** ..... / .... / ......

Name..Postcode............................

Date of birth .... / .... /......... Current Age..........

Current occupation...

How many children have you had: **0 1 2 3 4 5+** (please circle as appropriate).

Thinking back to the births, how many were:

- Normal vaginal deliveries...........
- Vaginal deliveries with forceps.........
- Vaginal deliveries with ventouse (suction)..........
- Caesarean section.............

---

2. Assessing Symptoms

How often do you leak urine? (please circle below)

Never Once a week or less 2-3 times a week once a day several times a day all the time

How much urine do you usually leak? (please circle below)

None a small amount a moderate amount a large amount

Overall, how much does leaking urine interfere with your everyday life? (please circle a number between 0 (not at all) and 10 (a great deal)

0 1 2 3 4 5 6 7 8 9 10

When does your urine leak?

- *Never – urine does not leak..........*
- *Leaks before you can go to the toilet..........*
- *Leaks when you cough or sneeze..........*
- *Leaks when you are asleep..........*
- *Leaks when you are physically active / exercising..........*
- *Leaks when you have finished urinating and are dressed...........*
- *Leaks for no obvious reason..........*
- *Leaks all the time..........*

....

3.Modified Oxford Score (to be completed by the practice nurse).

Please indicate the grading of the pelvic floor based on the Modified Oxford Scale by circling the appropriate number below:

0 = no contractions **1** = flicker **2** = weak **3** = moderate (with lift) **4** = good (with lift) **5** = strong (with lift)

4.Pelvic Floor Muscle Training Plan ***(only for those eligible)***

Here the practice nurse should briefly describe the care plan advised to the patient and how often the exercises should be carried out in line with the PERFECT scheme:

Exercise recommended

Intensity...

Number of Repetitions...

Number of Fast Contractions...

5.Follow Up

Have you recommended this patient for follow – up? Y/ N

If not, what further action? (i.e. patient refused to participate in exercises, or patient referred to other services.

………
………
………
…………………………………………………………

<u>6.Date to follow-up</u> (at least 3 months after the start of the exercise programme)………………………………….

Mode of follow up preferred: face to face ☐ or telephone ☐

(N.B: A face-to-face follow-up may provide an opportunity to measure any change to the MOS score).
