# Supplementary material for: Can primary care nurse administered pelvic floor muscle training (PFMT) be implemented for the prevention and treatment of urinary incontinence? A study protocol
Source: F1000Res. 2013 Feb 13;2:47. [Version 1] doi: 10.12688/f1000research.2-47.v1 (PMC3752732; doi:10.12688/f1000research.2-47.v1)
Supplement: Data collection sheets (baseline and follow up) — Data collection sheets that repeats the modified questions from the International Consultation on Incontinence Questionnaire (ICI-Q), completed at baseline and follow up [file f1000research-2-1176-s0000.tgz › Data_Collection_Sheet__follow_up_.pdf]

Data Collection Sheet (follow-up) – to be completed in consultation with the patient

CONFIDENTIAL

1. Background Information

Follow up Number .... Date of follow-up ...... / ...... / ......

Name .... Postcode ....

Date of birth .... / .... /.......... Current Age..........

Mode of follow-up: Face to Face ☐ Telephone ☐

Please insert your practice stamp

....

2. Assessing Symptoms

How often do you now leak urine? (please circle below)

Never Once a week or less 2-3 times a week once a day several times a day all the time

How much urine do you usually leak? (please circle below)

None a small amount a moderate amount a large amount

Overall, how much does leaking urine now interfere with your everyday life? (please circle a number between 0 (not at all) and 10 (a great deal)

0 1 2 3 4 5 6 7 8 9 10

When does your urine now leak?

- *Never – urine does not leak..........*
- *Leaks before you can go to the toilet..........*
- *Leaks when you cough or sneeze..........*
- *Leaks when you are asleep..........*
- *Leaks when you are physically active / exercising..........*
- *Leaks when you have finished urinating and are dressed...........*
- *Leaks for no obvious reason..........*
- *Leaks all the time..........*

3. Assessing Improvement

a) How often have you done your exercises (e.g. daily, twice daily etc.)?..

b) How did you remind yourself to do your exercises?
....
....
....

c) What did you find most helpful in reminding you to exercise?
....
....

d) Was there anything in particular that stopped you from doing your exercises?
....
....
....

e) Has there been an improvement in your urine leakage following your pelvic floor exercise plan? **Y / N / Not Applicable (if no previous symptoms)**

4. <u>Modified Oxford Score (if measured at follow-up to be completed by the Practice Nurse)</u>

Please indicate the grading of the pelvic floor based on the Modified Oxford Scale by circling the appropriate number below:

0 = no contractions **1** = flicker **2** = weak **3** = moderate (with lift) **4** = good (with lift) **5** = strong (with lift)

5. <u>Follow Up</u>

Have you recommended this patient for further follow – up? Y/ N

If not, what further action? (i.e. patient refused to participate in exercises, or patient referred to other services.

....
....
....
....

6. Date to follow-up ....

Mode of follow up preferred: face to face ☐ telephone ☐

(N.B. A face-to-face follow-up may provide an opportunity to measure any change to the MOS score).
