# Supplementary material for: Can primary care nurse administered pelvic floor muscle training (PFMT) be implemented for the prevention and treatment of urinary incontinence? A study protocol
Source: F1000Res. 2013 Feb 13;2:47. [Version 1] doi: 10.12688/f1000research.2-47.v1 (PMC3752732; doi:10.12688/f1000research.2-47.v1)
Supplement: Peer review history

# Peer review: Referee response for version 1

**Published:** 2013-02-20

**Authors:** Heinz Koelbl

**Affiliations:** Department of Obstetrics and Gynaecology, Johannes Gutenberg University, Mainz, Germany

I think this is an interesting study project, both for the primary aim of the study and for the good study design.It is surely of primary interest to evaluate if the postpartum PFMT, performed as a prevention therapy, can result in fewer referrals to secondary care for urinary incontinence (UI), and then reduce the number and associated costs of surgical procedures for UI.

The methodology planned to carry out the study is valid, and in particular the strategy of recruitment seems to be effective.

A few suggestions regarding the description of the future results:

- It would be necessary to stratify the population regarding the time after the last delivery (since the population enrolled is between 25-64 y, it would be interesting to understand if the PFMT performed just after the delivery, will be more effective than the PFMT executed later).
- More information regarding the PFMT is needed (e.g. for how long, only performed with trained nurse or performed autonomously after training).

The results of this trial will help surely to improve the clinical practice and management of UI patients.

I have read this submission. I believe that I have an appropriate level of expertise to confirm that it is of an acceptable scientific standard.

# Peer review: Referee response for version 1

**Published:** 2013-02-18

**Authors:** Roger Dmochowski

**Affiliations:** Department of Urology, Vanderbilt University, Nashville, TN, USA

I have read this submission. I believe that I have an appropriate level of expertise to confirm that it is of an acceptable scientific standard.
